# Predecessor and contemporary nursing: what is your legacy to future generations?

**DOI:** 10.1590/0034-7167-2024-0370

**Published:** 2025-06-20

**Authors:** Dirce Stein Backes, Betina Pereira Horbe, Marli Terezinha Stein Backes, Claudia Zamberlan

**Affiliations:** I Universidade Franciscana. Santa Maria, Rio Grande do Sul, Brazil; II Universidade Federal de Santa Catarina. Florianópolis, Santa Catarina, Brazil

**Keywords:** Nursing, Breeding, Time, Sustainable Development, Nonlinear Dynamics, Enfermería, Tiempo, Cruzamiento, Desarrollo Sostenible, Dinámicas No Lineales

## Abstract

**Objective:**

to reflect on the legacy of predecessor and contemporary nursing to future generations, with a view to sustainable development.

**Methods:**

Theoretical and reflective essay that considered the legacy of Nightingale, Anna Nery and Horta and contemporary bibliographical sources that reflect values and principles associated with sustainable development, in the light of complexity thinking.

**Results:**

The theoretical reflection focuses on four themes: Nightingale’s legacy - the forerunner of modern nursing; Anna Nery’s legacy - the heroine of Brazilian nursing; Horta’s legacy - the protagonist of the scientific foundations of nursing; and Contemporary nursing - what is its legacy to future generations?

**Final considerations:**

Recovering the legacy of Nightingale, Anna Nery and Horta is fundamental to advancing and sustaining the present and future of nursing. It is essential that contemporary nursing provokes disruptive movements and enables an evolutionary complex thinking that sustains health care as an interdependent, complementary and systemic phenomenon.

## RESUMEN

**Objetivo:**

Reflexionar acerca del legado de la enfermería predecesora y contemporánea, con vistas a un desarrollo sostenible.

**Métodos:**

Es un ensayo teórico-reflexivo basado en el legado de Nightingale, Anna Nery, Horta y fuentes bibliográficas contemporáneas incumbidas en valores y princípios asociados al desarrollo sostenible, bajo la luz del pensamiento de la complejidad.

**Resultados:**

La reflexion teórica está centrada en cuatro temas: legado de Nightingale - precursora de la enfermería moderna; legado de Anna Nery - heroína de la enfermería brasileña; legado de Horta - protagonista de los fundamentos científicos de la enfermería; Enfermería contemporánea - ¿cuál es su legado para las generaciones futuras?

**Consideraciones finales:**

Recuperar el legado de Nightingale, Anna Nery y Horta es esencial para avanzar y sostener el presente y el futuro de la enfermería contemporánea, promoviendo así movimientos disruptivos hacia un pensamiento complejo y evolutivo en la atención sanitaria como un fenómeno interdependiente, complementario y sistémico.

## INTRODUCTION

Climate change and environmental aggression are among the most serious threats to the dignity of human life and universal health. What a short time ago was considered a problem for the future is now showing signs of degradation and illness. Development, at any cost and without criteria, has deleterious consequences in terms of health, employment, access to resources, forced migration and other effects that denote ruptures and setbacks in public health^(1, 2)^.

At the same time, there are scholars who defend sustainable development in different areas of knowledge. Although its definition, history, pillars and principles remain unclear, sustainable development can/should be understood in its evolutionary process. In this process, we consider the human capacity to prospect, expand and advance in terms of complexity and social impact in order to creatively achieve progress with sustainability and well-being^(3)^. In this direction, nursing is recognized as an essential profession for promoting well-being and sustainable development, by contextualizing and conceiving care as a complex, complementary and interdependent unit^(4, 5)^.

It is therefore necessary to move forward without relegating to the background fundamental and inseparable values and principles that can be understood in the light of complexity thinking. This thinking incorporates the notion of sustainability and defends the importance of the universal and the particular, the singular and the multidimensional, the individual and the collective, the past and the present, from an evolutionary and prospective perspective. Systems (nursing, health and others) capable of interacting and situating themselves in this complex thinking will be capable of fruitfulness, survival and sustainability in human, social, political and planetary relationships^(6)^.

In recognizing and leveraging pillars of nursing’s past, present and future, and with a view to fostering more integrated, sustainable knowledge that is committed to citizenship, the question arises: What is the legacy of predecessor and contemporary nursing to future generations, with a view to sustainable development?

## OBJECTIVE

To reflect on the legacy of predecessor and contemporary nursing to future generations, with a view to sustainable development.

## METHODS

This is a theoretical-reflective essay that considered complex thinking as a theoretical and methodological contribution to the significance of the relevant findings. This essay was conceived by researchers who are part of the Nursing Social Entrepreneurship Research and Studies Group - GEPESES, specifically based on provocations and discussions related to the climate tragedy in southern Brazil.

In May 2024, torrential rains and floods devastated 90% of the state of Rio Grande do Sul. This tragedy was one of the biggest natural disasters in Brazilian history. In addition to the two million people directly affected and the hundreds of people missing, the tragedy destroyed roads, bridges and severely compromised human health and survival. Although Brazil occupies renewed diplomatic leadership at international level on environmental issues, government policies are not sufficiently clear and aligned with ecologically sustainable practices ^(7)^.

Thus, in order to bring about theoretical and practical advances, we sought out the legacy of Florence Nightingale^(8)^, Anna Nery^(9)^ and Wanda de Aguiar Horta^(10)^, as well as the contribution of contemporary nursing in promoting a healthy, sustainable environment that adds to human and social well-being. We used bibliographic sources by contemporary authors, available in the institutional library, who advocate complexity thinking to support nursing care as a complex, interdependent and inseparable unit of different social and environmental movements and phenomena. These bibliographic sources by contemporary authors were organized, analyzed and discussed by the researchers in the light of complex thinking.

The composition of this essay portrays connections between the undeniable past of the thinking and actions of nursing precursors Florence Nightingale, Anna Nery and Wanda Horta and their humanitarian and evolutionary attributes that continue to inspire contemporary nursing. It also seeks to demonstrate that Brazilian nursing has a strong commitment to sustainable development by (re)thinking theoretical and methodological references and by enabling broader discussions on environmental issues at Brazilian Nursing Congresses, among other alignments that consider climate change as a preponderant factor for human and planetary health^(11)^.

As this study did not use primary sources, it was not approved by the Research Ethics Committee. However, the ethical principles of scientific integrity were taken into account.

## RESULTS

The theoretical reflection focuses on four themes: Nightingale’s legacy - the forerunner of modern nursing; Anna Nery’s legacy - the heroine of Brazilian nursing; Horta’s legacy - the protagonist of the scientific foundations of nursing; and Contemporary nursing - what is its legacy to future generations?

### Legacy of Florence Nightingale - forerunner of modern nursing

Nightingale stood out for her strong initiative, determination and political influence in overcoming unhealthy and precarious contexts in terms of hygiene, sanitation and comfort. As well as conceiving and giving social and scientific value to nursing care, Nigthingale pushed his successors towards a new professional ideal, i.e. a new way of educating, advancing and persuading public leaders regarding the need for structural and organizational changes^(8)^.

Nightingale won international acclaim for her transformative leadership, her ability to overcome prejudices related to the activities carried out by women and her ability to supplant the assistentialist idea of health care. In her precariousness, Nightingale proclaimed human dignity, transcended biological aspects and achieved human well-being by defending environmental issues as essential to the care, promotion of health and healing of patients^(8)^.

Her determination for humanitarian and social causes drove Nightingale to care for wounded soldiers in the Crimean War (1853 to 1856) and to give up the noble social role she had inherited from her family. Far beyond reorganizing nursing and protecting lives during the war, Nightingale’s attributes of humanism, proactive leadership and political authority earned her a prominent place among the 100 most influential women in world history^(8)^.

### Anna Nery’s legacy - heroine of Brazilian nursing

Anna Nery, a pioneer of Brazilian nursing, was honored for her strong humanitarian spirit in caring for Paraguayan and Brazilian soldiers during the Paraguayan War in 1865, in which she voluntarily enlisted. She stood out for her visionary leadership in helping, caring for and providing health care to soldiers, as well as organizing an infirmary in her home during the campaign. She knew how to break paradigms, face adversity and promote care with dignity, humanity and ethics. For Anna Nery, health care was not reduced to the technical or biological dimension, but encompassed contexts and contours, taking into account natural resources such as medicinal plants, the environment and well-being in order to achieve comprehensive care and health for individuals and communities^(9)^.

A strong and fearless woman, Anna Nery knew how to create, take the lead, reinvent herself, promote the common good of the community and resist war even in the face of the death of her son. She challenged herself, was able to go beyond her own interests and became an inspiration to the women of her time and, for this reason, the first woman to occupy a seat in the Bahia Academy of Letters. She was sensitive to human and environmental pain and proved to be affable, innovative and transformative in various situations, especially in the face of the adversities of the time. Anna Nery had her name written in the Book of Heroes, which highlights personalities from Brazilian history^(9)^.

### Wanda de Aguiar Horta’s legacy - the protagonist of the scientific foundations of nursing

Recognized for her sensitivity, humanity and persistence, Wanda Horta became renowned among scientists and scholars for her autonomous and innovative scientific construction, for opening up paths, breaking down barriers and bringing about advances that had repercussions in different areas of knowledge. She distinguished herself by developing the Theory of Basic Human Needs. Similar to the protagonism of Nightingale and Anna Nery, Horta foresaw advances and transcended methods and approaches that disregarded environmental issues^(8)^.

As the protagonist of a thought that established the scientific foundations of Brazilian nursing, Wanda Horta went beyond her time and space. She knew how to dare, dialog, diverge, aggregate, lead and prospect a new theoretical-practical horizon - the Nursing Process with the characteristics of Brazilian nursing. Organized in cyclical, interdependent and recurring stages, the Nursing Process was conceived by Wanda Horta.

Nursing Process conceived by Wanda Horta transcends the theoretical-interventionist perception and reveals itself as an inductive and (re)ordering alternative for a new way of thinking and proceeding in relation to health care^(10, 12)^. In this inductive path, Wanda Horta made advances possible and led nursing to new scientific heights without neglecting ethical, social and environmental issues related to sustainable development.

### Contemporary nursing - what is its legacy to future generations?

Contemporary nursing, as the protagonist and driving force behind expanded, contextualized and multidimensional health care, is driven to consolidate its humanitarian and sustainable attributes. It is also compelled to confront the disorders resulting from health crises (pandemics and epidemics), climate change (excessive rainfall, floods, extreme heat, droughts, among others) and ecologically irresponsible practices that degrade human, social and political dignity and, consequently, natural and environmental resources. Through good sustainable practices, nursing professionals can easily adapt to adverse situations in order to be resolute in referrals, optimize the work process and minimize unnecessary travel and interventions, among other things^(10, 11, 12, 13)^.

The current historical moment, aggravated by climate change, calls for coherence in life, human, social and ethical relations, an apprehension of human singularities in the face of contradictions and segregations, and participatory, inclusive and responsible leadership. Considered one of the professions most engaged in health promotion, nursing is called upon to combat social and gender inequalities, to defend the construction of more equitable and peaceful societies, to contribute to reducing excessive spending on health, to induce emancipatory processes capable of shifting individual thinking towards collective and collaborative action, in favor of a fairer, more prosperous and sustainable world for present and future generations.

It is imperative that nursing considers sustainable development in undergraduate and postgraduate curricula and develops mechanisms related to environmental education and the development of theoretical and practical approaches that consider the inseparability of health care^(10)^. However, what is the legacy of contemporary nursing for the present and future generations, given the fragmentation of knowledge and practices that operate either by simplification or reproduction?

## DISCUSSION

Faced with climate change and its consequent devastation and setbacks, such as the event that took place in Rio Grande do Sul in May 2024, it is essential to intensify processes, behaviors and a driving thought that leads to sustainable development. It is also essential to understand health care as a complex unit, interdependent and interconnected with the various sectors. There is an urgent need to relearn sustainable and humanitarian behaviors and practices advocated by nursing’s predecessors, whose legacy remains alive and inspiring, even in other areas of knowledge.

We are therefore in a complex situation, which requires equally complex intersectoral and interprofessional responses and advances. Complexity thinking emerges, under this approach, with hologrammatic movements, propelling systemic paths fragmented by disjunctive and utilitarian thinking. Complexity thinking emerges as an integrating phenomenon capable of organizing, analyzing and “weaving together” the agents that promote sustainable development^(6)^.

By questioning the predictable, the absolute and the linearity of human actions, Morin (one of the protagonists of complexity thinking) advocates a systemic alignment, capable of questioning the limits of science and the mutilating mind that apprehends only a part to satisfy local and transitory interests. Morin makes it possible to rethink planetary reality, based on contextualization and the apprehension of the singular and the multidimensional, the local and the global, the part and the whole and vice versa, with a view to sustainable development^(6, 14)^.

Understanding the world in its prospective, evolutionary and sustainable dynamics, in the light of complexity thinking, necessarily requires open, flexible and adaptable minds, capable of contextualizing, confronting and interconnecting facts and events in a multidimensional and systemic way. The nurse with a well-made mind, in the logic of complexity thinking, is capable of conceiving a new way of perceiving, dynamizing and prospecting evolutionary and sustainable movements, like their predecessors, based on self-reflection, self-awareness and the propulsion of interdependent processes and paths^(15)^.

Sustainable development therefore induces thinking autonomy, the conscious and rational use of natural resources, political protagonism that considers human and social interrelationships, the promotion of collegiate, participatory and systemic paths in the different areas of knowledge^(16)^. Under this impulse, contemporary nursing is expected to adopt a new way of being, thinking and living in community, courage to review choices and positions, initiative to induce disruptive movements and leadership to bring together and prospect human and social processes that involve solidarity, cooperation, respect and tolerance.

Everything and everyone is interconnected and, collaboratively, challenged and called upon to assume renewed human and professional attitudes. So, based on the legacy of Nightingale, Anna Nery and Horta, what should we change, what should we keep, where and how should we evolve in a sustainable way in the field of nursing and health? The existence and meaning of human beings and society are deeply associated with questions of the sustainability of life, care, health and the environment. From this perspective, sustainable development promotes the inseparable dynamics between the environmental, social and economic systems, based on actions and mechanisms for conserving and promoting quality of life for current and future generations^(17)^.

Complexity thinking must be apprehended and understood as a fabric of inseparably associated heterogeneous elements, which represent the paradoxical relationship between the one and the multiple, between the past, present and future^(14)^. This thinking is capable of associating and interconnecting knowledge that is apparently dispersed, disorganized and relegated to the background, such as the humanitarian legacy of the pioneers of nursing.

Sustainable development implies, on the part of contemporary nursing and the community in general, overcoming the current technocratic model, rethinking attitudes, behaviours and combining development in such a way as to harmonize social, environmental and economic objectives. The notion of sustainability implies reviewing the possibilities for growth and prospecting strategies that take into account human-social interlocutions through educational practices and dialogic processes that favor interconnectivity and collegiate and collaborative work^(10)^.

### Limitations of the study

A limitation of this study is that it did not use a validated methodological framework to support the theoretical and reflective design. Another limitation is associated with the synthetic description of the legacy of the three predecessors, given their vast historical legacy to contemporary nursing. It does, however, provide prospective reflections for other nursing thinkers/researchers to advance in the deliberation of new perspectives that induce sustainable development in contemporary times.

### Contributions to the field of nursing and health

The contributions of this study to the advancement of Brazilian nursing science are associated with the realization that contemporary nursing has a history, a legacy and social recognition with the potential to inspire professionals at the international level in terms of achieving the Sustainable Development Goals. Another contribution is associated with the proposition of evolutionary thinking and sustainable actions among nursing professionals, in order to overcome care practices, fragmented behaviors and irresponsible attitudes in the face of growing climate change that demand a new way of thinking and acting in society.

## FINAL CONSIDERATIONS

Recovering the legacy of Nightingale, Anna Nery and Horta is fundamental to moving forward, but not enough to sustain the present and future of nursing. It is essential that contemporary nursing provokes disruptive movements and enables an evolutionary complex thinking that sustains health care as an interdependent, complementary and systemic phenomenon.

To reflect on the legacy of nursing’s predecessors is to reaffirm the social value of this profession and reinvigorate its essential role in promoting healthy and sustainable environments. It is hoped that the legacy left by these great women will serve as inspiration and encouragement for the nurses of today and the future as protagonists of improvements in public health around the world.
