# Supplementary material for: Predecessor and contemporary nursing: what is your legacy to future generations?
Source: Rev Bras Enferm. 2025 Jun 20;78(Suppl 1):20240370. doi: 10.1590/0034-7167-2024-0370 (PMC12185116; doi:10.1590/0034-7167-2024-0370)
Supplement: Translation

# Translation: Enfermagem predecessora e contemporânea: qual o seu legado às gerações futuras?

**DOI:** 10.1590/0034-7167-2024-0370pt

**Authors:** Dirce Stein Backes, Betina Pereira Horbe, Marli Terezinha Stein Backes, Claudia Zamberlan

**Affiliations:** I Universidade Franciscana. Santa Maria, Rio Grande do Sul, Brasil; II Universidade Federal de Santa Catarina. Florianópolis, Santa Catarina, Brasil

**Keywords:** Enfermagem, Geração, Tempo, Desenvolvimento Sustentável, Dinâmica Não Linear

## Abstract

**Objetivo:**

Refletir sobre o legado da enfermagem predecessora e contemporânea às gerações futuras, com vistas ao desenvolvimento sustentável.

**Métodos:**

Ensaio teóricoreflexivo que considerou o legado de Nightingale, Anna Nery e Horta e fontes bibliográficas contemporâneas que refletem valores e princípios associados ao desenvolvimento sustentável, à luz do pensamento da complexidade.

**Resultados:**

A reflexão teórica centra-se em quatro temáticas: legado de Nightingale - precursora da enfermagem moderna; legado de Anna Nery - heroína da enfermagem brasileira; legado de Horta - protagonista das bases científicas de enfermagem e Enfermagem contemporânea - qual o seu legado às gerações futuras?

**Considerações finais:**

Recuperar o legado de Nightingale, Anna Nery e Horta é fundamental para avançar e sustentar o presente e o futuro da enfermagem. É imprescindível que a enfermagem contemporânea provoque movimentos disruptivos e possibilite um pensamento complexo evolutivo que sustente o cuidado em saúde como fenômeno interdependente, complementar e sistêmico.

## INTRODUÇÃO

As alterações climáticas e a agressão ao meio ambiente estão entre as ameaças mais graves à dignidade da vida humana e à saúde universal. O que há pouco tempo era considerado um problema para o futuro, hoje, evidencia sinais de degradação e adoecimento. O desenvolvimento, a qualquer custo e sem critérios, apresenta consequências deletérias em âmbito da saúde, emprego, acesso a recursos, migrações forçadas e outros efeitos que denotam rupturas e retrocessos à saúde pública^(1, 2)^.

Evidencia-se, paralelamente, estudiosos que defendem o desenvolvimento sustentável nas diferentes áreas do conhecimento. Embora a sua definição, história, pilares e princípios permanecem pouco claros, o desenvolvimento sustentável pode/deve ser apreendido em seu processo evolutivo. Considera-se, nesse percurso, a capacidade humana de prospectar, ampliar e avançar em termos de complexidade e impacto social para alcançar, criativamente, avanços com sustentabilidade e bem-estar^(3)^. Nessa direção, a enfermagem é reconhecida como profissão essencial à promoção do bem-estar e do desenvolvimento sustentável, ao contextualizar e conceber o cuidado como unidade complexa, complementar e interdependente^(4, 5)^.

É preciso, portanto, avançar sem relegar a um segundo plano valores e princípios fundantes e indissociáveis que podem ser compreendidos à luz do pensamento da complexidade. Esse pensamento incorpora a noção de sustentabilidade e defende a importância do universal e do particular, do singular e do multidimensional, do individual e do coletivo, do passado e do presente, a partir de uma perspectiva evolutiva e prospectiva. Os sistemas (enfermagem, saúde e outros) capazes de interagir e situar-se neste pensamento complexo estarão aptos à fecundidade, à sobrevivência e à sustentabilidade dos relacionamentos humanos, sociais, políticos e planetários^(6)^.

Ao reconhecer e potencializar pilares do passado, presente e futuro da enfermagem e com vistas ao fomento de um saber mais integrado, sustentável e comprometido com a cidadania, questiona-se: Qual o legado da enfermagem predecessora e contemporânea às gerações futuras, com vistas ao desenvolvimento sustentável?

## OBJETIVO

Refletir sobre o legado da enfermagem predecessora e contemporânea às gerações futuras, com vistas ao desenvolvimento sustentável.

## MÉTODOS

Trata-se de um ensaio teórico-reflexivo que considerou o pensamento complexo como aporte teórico e metodológico para a significação dos achados pertinentes. O presente ensaio foi idealizado por pesquisadores que integram o Grupo de Pesquisa e Estudos em Empreendedorismo Social da Enfermagem - GEPESES, mais especificamente a partir de provocações e discussões relacionadas à tragédia climática do sul do Brasil.

Em maio de 2024, chuvas torrenciais e inundações devastaram 90% do Estado do Rio Grande do Sul. Essa tragédia foi um dos maiores desastres naturais da história do Brasil. Além de duas milhões de pessoas diretamente atingidas e das centenas de pessoas desaparecidas, a tragédia destruiu estradas, pontes e comprometeu severamente a saúde e a sobrevivência humana. Embora o Brasil ocupe renovada liderança diplomática em âmbito internacional no que se refere às questões ambientais, as políticas governamentais não estão suficientemente claras e alinhadas às práticas ecologicamente sustentáveis^(7)^.

Assim, no intuito de provocar avanços teórico-práticos, buscou-se o legado de Florence Nightingale^(8)^, Anna Nery^(9)^ e Wanda de Aguiar Horta^(10)^, além da contribuição da enfermagem contemporânea na promoção de ambiência saudável, sustentável e agregadora de bem-estar humano e social. Utilizou-se fontes bibliográficas de autores contemporâneos, disponíveis na biblioteca institucional, que defendem o pensamento da complexidade para sustentar o cuidado de enfermagem como unidade complexa, interdependente e indissociável dos diferentes movimentos e fenômenos sociais e ambientais. Essas fontes bibliográficas de autores contemporâneos foram organizadas, analisadas e discutidas pelos pesquisadores, à luz do pensamento complexo.

Retrata-se, na composição deste ensaio, conexões entre um passado inegável do pensar e agir das precursoras de enfermagem Florence Nightingale, Anna Nery e Wanda Horta e os seus atributos humanitários e evolutivos que seguem inspirando a enfermagem contemporânea. Procura-se, ainda, demonstrar que a enfermagem brasileira tem forte compromisso com o desenvolvimento sustentável ao (re)pensar referenciais teórico-metodológicos e ao possibilitar discussões ampliadas sobre as questões ambientais em Congressos Brasileiros de Enfermagem, dentre outros alinhamentos que consideram as alterações climáticas como fator preponderante à saúde humana e planetária^(11)^.

Pelo fato deste estudo não utilizar fontes primárias, o mesmo não passou pelo Comitê de Ética em Pesquisa. Considerou-se, contudo, os princípios éticos da integridade científica.

## RESULTADOS

A reflexão teórica centra-se em quatro temáticas: legado de Nightingale - precursora da enfermagem moderna; legado de Anna Nery - heroína da enfermagem brasileira; legado de Horta -protagonista das bases científicas de enfermagem e Enfermagem contemporânea - qual o seu legado às gerações futuras?

### Legado de Florence Nightingale – precursora da enfermagem moderna

Nightingale distinguiu-se pela sua forte iniciativa, determinação e influência política na transposição de contextos insalubres e precários em termos de higiene, saneamento e conforto. Além de conceber e imprimir valor social e científico ao cuidado de enfermagem, Nigthingale impulsionou os seus sucessores a um novo ideal profissional, ou seja, a um novo modo de educar, avançar e persuadir lideranças públicas no que se refere às necessidades de mudanças estruturais e organizacionais^(8)^.

Nightingale se condecorou, em âmbito internacional, pela sua liderança transformadora, capacidade de superar preconceitos relacionados às atividades exercidas pelas mulheres e de suplantar a ideia assistencialista do cuidado em saúde. Na precariedade, Nightingale proclamou a dignidade humana, transcendeu aspectos biológicos e alcançou o bem-estar humano ao defender as questões ambientais como essenciais ao cuidado, à promoção da saúde e à cura dos pacientes^(8)^.

A determinação pelas causas humanitárias e sociais impulsionaram Nightingale a atender os soldados feridos na Guerra da Criméia (1853 a 1856) e a abdicar-se de um papel social de nobreza que herdara de sua família. Muito além de reorganizar a enfermagem e proteger vidas durante a guerra, Nightingale se projetou, no cenário mundial, pelos seus atributos humanismo, liderança proativa e autoridade política, às quais lhe possibilitaram um lugar de destaque entre as 100 mulheres mais influentes da história mundial^(8)^.

### Legado de Anna Nery – heroína da enfermagem brasileira

Anna Nery, pioneira da enfermagem brasileira, foi engrandecida pelo seu forte espírito humanitário no cuidado aos soldados paraguaios e brasileiros por ocasião da Guerra do Paraguai, em 1865, na qual se alistou voluntariamente. Destacou-se pela sua liderança visionária no socorro, cuidado e atendimento de saúde aos soldados, bem como na organização de uma enfermaria em sua casa na Campanha. Soube romper paradigmas, enfrentar adversidades e promover o cuidado com dignidade humanidade e ética. O cuidado em saúde, para Anna Nery, não se reduziu à dimensão técnica ou biológica, mas abarcou contextos e contornos, ao considerar os recursos naturais como as plantas medicinais, o meio ambiente e o bem-estar para alcançar o cuidado e a saúde integral dos indivíduos e coletividades^(9)^.

Mulher forte e destemida, Anna Nery soube criar, protagonizar, reinventar-se, promover o bem-comum da coletividade e resistir à guerra mesmo diante da morte de seu filho. Desafiou-se, foi capaz de ir além dos próprios interesses e tornou-se inspiração para as mulheres de seu tempo e, por isso, a primeira mulher a ocupar uma cadeira na Academia de Letras da Bahia. Foi sensível às dores humanas e ambientais e mostrou-se afável, inovadora e transformadora em diversas situações, sobretudo, face às adversidades da época. Anna Nery teve o seu nome escrito no livro dos heróis, o qual destaca personalidades da história brasileira^(9)^.

### Legado de Wanda de Aguiar Horta – protagonista das bases científicas de enfermagem

Reconhecida pela sua sensibilidade, humanidade e persistência, Wanda Horta tornou-se reconhecida entre cientistas e estudiosos, pela sua construção científica autônoma e inovadora, por abrir caminhos, romper barreiras e provocar avanços que repercutiram nas diferentes áreas do conhecimento. Distinguiu-se pelo desenvolvimento da Teoria das Necessidades Humanas Básicas. Semelhante ao protagonismo de Nightingale e Anna Nery, Horta prospectou avanços e transcendeu métodos e abordagens que desconsideravam as questões ambientais^(8)^.

Como protagonista de um pensamento que instaurou as bases científicas da enfermagem brasileira, Wanda Horta foi além de seu tempo e espaço. Soube ousar, dialogar, divergir, agregar, liderar e prospectar um novo horizonte teórico-prático - o Processo de Enfermagem com as características da enfermagem brasileira. Organizado em etapas cíclicas, interdependentes e recorrentes, o

Processo de Enfermagem concebido por Wanda Horta transcende a percepção teórico-intervencionista pontual e se revela como alternativa indutiva e (re)ordenadora de um novo modo de pensar e proceder em relação ao cuidado em saúde^(10, 12)^. Nesse percurso indutivo, Wanda Horta viabilizou avanços e conduziu a enfermagem a novos patamares científicos sem descuidar das questões éticas, sociais e ambientais relacionadas ao desenvolvimento sustentável.

### Enfermagem contemporânea – qual o seu legado às gerações futuras?

A enfermagem contemporânea, enquanto protagonista e agente propulsora do cuidado ampliado, contextualizado e multidimensional em saúde, é impulsionada a consolidar os atributos humanitários e sustentáveis. É impelida, igualmente, a fazer frente às desordens decorrentes das crises sanitárias (pandemia e epidemias), mudanças climáticas (chuvas em excesso, enchentes, calor extremo, secas, entre outros) e de práticas ecologicamente irresponsáveis que degradam a dignidade humana, social, política e, consequentemente, os recursos naturais e ambientais. Os profissionais de enfermagem, por meio de boas práticas sustentáveis, facilmente adaptam-se às situações adversas, de modo a serem resolutivos nos encaminhamentos, a otimizarem o processo de trabalho e a minimizarem deslocamentos e intervenções desnecessárias e outros^(10, 11, 12, 13)^.

O momento histórico atual, agravado pelas alterações climáticas, induz à coerência de vida, às relações humanas, sociais e éticas, à apreensão das singularidades humanas face as contradições e segregações, à liderança participativa, inclusiva e responsável. Considerada uma das profissões mais engajadas na promoção da saúde, a enfermagem é chamada a combater as desigualdades sociais e de gênero, a defender a construção de sociedades mais equitativas e pacíficas, a contribuir para a redução dos gastos excessivos em saúde, a induzir processos emancipatórios capazes de deslocar o pensamento individual ao agir coletivo e colaborativo, em prol de um mundo mais justo, próspero e sustentável às gerações presentes e futuras.

É premente que a enfermagem considere o desenvolvimento sustentável nos currículos de graduação e pós-graduação e desenvolva mecanismos relacionados à educação ambiental e o desenvolvimento de abordagens teórico-práticas que considerem a indissociabilidade do cuidado em saúde^(10)^. No entanto, qual o legado da enfermagem contemporânea para o presente e as gerações futuras, face à fragmentação de saberes e práticas que operam ora por simplificação, ora por reprodução?

## DISCUSSÃO

Face às mudanças climáticas e as suas consequentes devastações e retrocessos, a exemplo do evento ocorrido no Rio Grande do Sul, em maio de 2024, é inadiável intensificar processos, condutas e um pensamento propulsor que conduza ao desenvolvimento sustentável. É essencial, igualmente, apreender o cuidado em saúde como unidade complexa, interdependente e interligado aos diversos setores. É premente reaprender condutas e práticas sustentáveis e humanitárias defendidas pelas predecessoras de enfermagem, cujo legado permanece vivo e inspirador, inclusive, em outras áreas do conhecimento.

Encontramo-nos, portanto em uma engrenagem complexa, que requer respostas e avanços intersetoriais e interprofissionais igualmente complexas. O pensamento da complexidade emerge, sob esse enfoque, com movimentos hologramáticos, propulsores de percursos sistêmicos fragmentados pelo pensamento disjuntivo e utilitarista. O pensamento da complexidade emerge como fenômeno integrador capaz de organizar, analisar e “tecer em conjunto” os agentes que promovem o desenvolvimento sustentável^(6)^.

Ao questionar o previsível, o absoluto e a linearidade das ações humanas, Morin (um dos protagonistas do pensamento da complexidade) defende um alinhamento sistêmico, capaz de questionar os limites da ciência e a mente mutilante que apreende apenas uma parte para satisfazer os interesses locais e transitórios. Morin oportuniza repensar a realidade planetária, a partir da contextualização e da apreensão do singular e do multidimensional, do local e do global, da parte e do todo e vice-versa, com vistas ao desenvolvimento sustentável^(6, 14)^.

Apreender o mundo em sua dinâmica prospectiva, evolutiva e sustentável, à luz do pensamento da complexidade, requer, necessariamente, mentes abertas, flexíveis e adaptáveis, capazes de contextualizar, confrontar e interconectar os fatos e acontecimentos de forma multidimensional e sistêmica. O enfermeiro detentor de cabeça bem-feita, na lógica do pensamento da complexidade, é capaz de conceber um novo modo de perceber, dinamizar e prospectar movimentos evolutivos e sustentáveis, a exemplo das predecessoras, a partir da autorreflexão, da autoconscientização e da propulsão de processos e percursos interdependentes^(15)^.

O desenvolvimento sustentável induz, portanto, à autonomia pensante, ao uso consciente e racional dos recursos naturais, ao protagonismo político que considera as interrelações humanas e sociais, à promoção de percursos colegiados, participativos e sistêmicos nas diferentes áreas do conhecimento^(16)^. Espera-se da enfermagem contemporânea, sob esse impulso, um novo modo de ser, pensar e conviver em comunidade, coragem para rever escolhas e posicionamentos, iniciativa para induzir movimentos disruptivos e liderança para congregar e prospectar processos humanos e sociais que envolvem a solidariedade, a cooperação, o respeito e a tolerância.

Tudo e todos estamos interconectados e, colaborativamente, interpelados e convocados a assumir renovadas posturas humanas e profissionais. Assim, com base no legado de Nightingale, Anna Nery e Horta o que mudar, o que manter, onde e como evoluir de forma sustentável no campo da enfermagem e saúde? A existência e o significado do ser humano e da sociedade estão profundamente associados às questões de sustentabilidade da vida, do cuidado, da saúde, do ambiente. Sob esse enfoque, o desenvolvimento sustentável promove a dinâmica indissociável entre o sistema ambiental, social e econômico, a partir de ações e mecanismos de conservação e promoção da qualidade de vida às atuais e futuras gerações^(17)^.

O pensamento da complexidade deve ser apreendido e compreendido como um tecido de elementos heterogêneos inseparavelmente associados, que representam a relação paradoxal entre o uno e o múltiplo, entre o passado, presente e futuro^(14)^. Esse pensamento é capaz de associar e interligar saberes aparentemente dispersos, desordenados e relegados a um segundo plano, como, por exemplo, o legado humanitário das precursoras de enfermagem.

O desenvolvimento sustentável implica, por parte da enfermagem contemporânea e comunidade em geral, suplantar o modelo tecnocrático vigente, repensar atitudes, comportamentos e conjugar o desenvolvimento de modo a harmonizar os objetivos sociais, ambientais e econômicos. A noção de sustentabilidade implica em rever as possibilidades de crescimento e prospectar estratégias que levem em conta as interlocuções humano-sociais por meio de práticas educativas e de processos dialógicos que favoreçam a interconectividade e o trabalho colegiado e colaborativo^(10)^.

### Limitações do estudo

Considera-se como limitação deste estudo a não utilização de um referencial metodológico validado de suporte para o delineamento teórico-reflexivo. Outra limitação está associada à descrição sintética do legado das três predecessoras face ao seu vasto legado histórico à enfermagem contemporânea. Possibilita-se, no entanto, reflexões prospectivas para que outros pensadores/investigadores de enfermagem avancem na deliberação de novas perspectivas que induzam o desenvolvimento sustentável na contemporaneidade.

### Contribuições para a área de enfermagem e saúde

As contribuições deste estudo para o avanço da ciência brasileira de enfermagem estão associadas à percepção de que a enfermagem contemporânea tem uma história, um legado e um reconhecimento social com potencial para inspirar profissionais em âmbito internacional no que ser refere ao alcance dos Objetivos do Desenvolvimento Sustentável. Outra contribuição está associada à proposição de um pensamento evolutivo e ações sustentáveis entre os profissionais de enfermagem, no sentido de superar práticas assistencialistas, comportamentos fragmentados e atitudes irresponsáveis face às crescentes alterações climáticas que demandam um novo modo de pensar e agir na sociedade.

## CONSIDERAÇÕES FINAIS

Recuperar o legado de Nightingale, Anna Nery e Horta é fundamental para avançar, mas não o suficiente para sustentar o presente e o futuro da enfermagem. É imprescindível que a enfermagem contemporânea provoque movimentos disruptivos e possibilite um pensamento complexo evolutivo que sustente o cuidado em saúde como fenômeno interdependente, complementar e sistêmico.

Refletir sobre o legado das predecessoras de enfermagem é reafirmar o valor social dessa profissão e revigorar o seu papel essencial na promoção de ambientes saudáveis e sustentáveis. Espera-se que a herança deixada por essas grandes mulheres sirva de inspiração e encorajamento para os enfermeiros de hoje e do futuro como protagonistas de melhorias na saúde pública em todo o mundo.
